# A Great Reformation Needed: A Call to Duty

**Published:** 1908-03

**Authors:** Alexander S. Garrett

**Affiliations:** Springtown, Texas


For the Texas Medical Journal.

### A Great Reformation Needed: A Call to Duty.

BY ALEXANDER S. GARRETT, M. D., SPRINGTOWN, TEXAS.

It is patent to all reading, thinking, observing physicians that we are in the beginning of one of the greatest and most important social reformations ever known in the history of the world. Just how, where and when it started I am not prepared to say. Whether the reformation first originated in France under or by the suggestion and leadership of Professor Fornier in 1900, as I have seen stated, or whether the spirit of reformation touched the hearts of others in this country at the same time, before or since, I know not. But I do know that the reformation is now at hand, and that it is knocking at the hearts and consciences af every lover of humanity within the pale of the medical profession who is aware of its presence, and of the need of a reformation in regard to the great "social evil," or venereal diseases.

The curse and scourge of gonorrhea and syphilis have become so great that the medical profession must remove the veil of darkness and ignorance, expose the danger, the rottenness, the awful results and "cry aloud and spare not." The physician must be as a watchman on the walls of Jerusalem, who "will not hold his peace day nor night" till humanity is freed and redeemed from the pollutions and ravaging effects of venereal diseases.

Adding the experiences, observations and testimonies of the general practitioner in the country and small towns to the ponderous and fearful observations and testimonies of the surgeons, specialist and hospital physicians of the large cities, we are astounded and confounded with the awful havoc the social evil is playing with the husbands and wives and sons and daughters of the human race all over the inhabited world.

It may be true that a large number of physicians, especially those who graduated many years ago, and have not kept altogether in touch with the more recent observations and teachings, more especially about gonorrhea, do not realize the magnitude of the social reformation. To that class, if there be any, let me say, in all seriousness and earnestness, the battle cry has gone out. Fall into line! Physicians everywhere are to use all the knowledge and influence they have and can bring to bear to save or help save our nation from the awful and degenerating effects in so many ways of syphilis and gonorrhea.

Syphilis and gonorrhea are becoming so common that a pure, innocent, virtuous woman who enters into the holy bonds of matrimony may be entering into a union by which she is to receive an infection from her own husband, unknowingly and unintentionally it no doubt may be on his part, which will make her an unhappy, miserable, suffering invalid the balance of her days.

Dr. Prince A. Morrow, one of the greatest, if not the greatest or foremost, leader in the reform movement, says: "It may be a startling statement, but, nevertheless, true, that there is more venereal infection among virtuous wives than professional prostitutes in this country." Doctors, we do not know ourselves, when our daughters get married, especially if they marry a town man or city man, whether he is truly a well man or not. What of an uninformed public?

From 40 to 60 per cent of all inflammations of the womb and its peritoneal coverings are said to be of gonorrheal origin. Add to this the loathing spectacle of syphilis in all its horrid forms, the sorrowful disappointments and sufferings of mothers in abortions, miscarriages and premature deaths caused by syphilis, and we have a social state of affairs that make us shudder to contemplate.

Add to this the appalling fact that a large per cent of childless marriages are the result of gonorrhea contracted by the husband before marriage, and we shudder still more to contemplate the defilement of our social conditions.

While the great surgeons and specialists tell us about these conditions, in large numbers and in large per cents, there are very few general practitioners who do not know something of the awfulness and terribleness of these diseases in a more limited sense.

When we add to the above conditions the strictures, rheumatisms and various other diseases resulting therefrom, together with the social unhappiness in many ways, we are made to wonder why the medical profession has been so long finding out these awful facts.

Some years ago the writer practiced medicine in a small town in Texas where he was informed that a physician who had preceded him several years before, actually imported a woman with gonorrhea for the purpose of increasing his practice. Strange to say, it was said the "good" doctor was one of the first patients with gonorrhea! Had he known of the remote effects of gonorrhea, leaving out the sin, he would never have committed such an act.

Gonorrhea is on the increase. It must be checked.

A young medical student who was at home in our town during the Christmas holidays from one of our Texas medical colleges told the writer that by far the largest per cent of clinical cases in his college were venereal.

A druggist in our town, who was formerly in the drug business in one of our largest cities, says that it was no unusual occurrence to fill prescriptions for boys 12 to 14 years old for gonorrhea.

At the meeting of the State Medical Association at Mineral Wells, last year, I had prepared some resolutions to introduce before the House of Delegates, looking towards the education of the boys and girls in our public schools on the nature of venereal diseases. After conferring with several prominent delegates, I was persuaded not to offer the resolutions at that time.

I want to conclude this paper by quoting from ex-Governor W. J. Northen, of Georgia, in a recent address before the Atlanta Society for Sanitary and Moral Prophylaxis. He said: "If these things are undermining our social fabric to the alarming extent repeatedly stated in this presence this evening, I deliberately charge that you will be criminal before God and the people of our city if you do not have all of us know to the fullest extent, that you know, the dangers that confront us, because of the social sin. If what you said is true, and I do not doubt one word of your utterances, there ought to be great and glaring red lights before the eyes of every man in Atlanta, including all classes, so that all of us may know and apply the remedies, before destructive evils thoroughly poison our whole social system." * * * "If the so-called social evil is so destructive to the young, why not make proper instructions upon this subject a part of our school system, and have the younger generation of both sexes, fully informed as to the injurious effects that follow impurity and moral uncleanness that seem to be so common over the land? With these evils so alarmingly prevalent and so destructive of mind, body and spirit, we can but be cruelly unkind if we do not thoroughly inform the youth of the city, before they shall be ignorantly destroyed.

"When you say that 30 or 40 per cent of the blindness among the unfortunates of this class of our people is to be attributed to the destructive forces of the so-called social evil, how can we delay to inform men, lest we allow—purposely allow—the ruin of the race and approve the open shame of the home?

"When you tell me that 50 to 75 per cent of the male population of the larger cities is more or less afflicted with this moral leprosy, you challenge my faith in the people if I retain my confidence in your statement and my regard for your opportunities to know.

"All things being true, as your discussion must lead us to believe, I charge you, in the name of clean living and in the name of humanity that seems to be going steadily and rapidly to decay, lay aside false modesty, call these dreadful things by their horrid names, and, with heroic effort, begin the recovery of the generation through the purity of individual life and the sanctity and virtue of the homes of all the people.

"By all means, do not let the matter remain as it now is, but call another and a larger meeting and tell again all you have said tonight to all the people, who can be induced to come. We should speedily redeem the city from the curse of this sin."—Quoted from the *Atlanta Journal-Record of Medicine*, January, 1908.

In the *Texas Medical News*, September, 1905, under the head of "The Relation of the Physician to the Community," I called the attention of the medical profession to the alarming extent of this monster evil.

I come again in the name of God and the cause of humanity and ask every physician who may read this paper to sound the tocsin of alarm from the office, the streets, at the home, in the Young Men's Christian Associations, or anywhere under the canopy of heaven where a proper word of warning and admonition can be given.

